# TUBA1B as a novel prognostic biomarker correlated with immunosuppressive tumor microenvironment and immunotherapy response

**DOI:** 10.3389/fphar.2025.1517887

**Published:** 2025-02-04

**Authors:** Juntao Qi, Mingming Zhou, Na Yang, Huiyun Ma, Min He, Gujie Wu, Chang Ge, Liuyin Jin, Lin Cheng, Wei Liao, Hefei Ren, Caiyun Lei

**Affiliations:** ^1^ Rehabilitation Medicine Department, Hunan Aerospace Hospital, Hunan Normal University, Changsha, Hunan, China; ^2^ Research Center of Clinical Medicine, Shenzhen Hospital of Shanghai University of Traditional Chinese Medicine, Shenzhen, China; ^3^ Department of Critical Care Medicine, Chongqing University Affiliated Cancer Hospital, Chongqing, China; ^4^ Laboratory of Oncology and Immunology, School of Basic Medical Sciences, Guangdong Pharmaceutical University, Guangzhou, China; ^5^ Research Center of Clinical Medicine, Affiliated Hospital of Nantong University, Nantong, China; ^6^ Shanghai Medical College, Fudan University, Shanghai, China; ^7^ School of Medicine, Wuhan University, Wuhan, China; ^8^ Department of Otolaryngology and Head and Neck Surgery, The Fifth Affiliated Hospital of Sun Yat-sen University, Zhuhai, Guangdong, China; ^9^ Department of Laboratory Medicine, Changzheng Hospital, Naval Medical University, Shanghai, China

**Keywords:** TUBA1B, prognosis, biomarker, tumor microenvironment, anti-tumor strategies

## Abstract

**Background:** Tubulin alpha 1b (TUBA1B) is a key microtubule protein essential for maintaining cellular structure and function. This protein contributes significantly to cytoskeletal formation and is implicated in various diseases. Despite its fundamental roles, TUBA1B’s impact on tumor prognosis and the tumor immune microenvironment across cancer types remains inadequately understood.

**Methods** To elucidate TUBA1B’s role in cancer prognosis and immune response, we conducted a comprehensive analysis, integrating data from established databases such as The Cancer Genome Atlas, Genotype Tissue Expression, Cancer Cell Lineage Encyclopedia, Human Protein Atlas, Kaplan-Meier Plotter, cBioPortal, TIMER, and ImmuCellAI, along with a large-scale clinical study and immunotherapy cohort. We also conducted *in vitro* functional assays to assess TUBA1B’s functional role in tumor cells, allowing for a detailed examination of its relationship with cancer prognosis and immune modulation.

**Results:** Our findings indicate that TUBA1B expression is dysregulated across multiple cancers, correlating strongly with poor survival outcomes and advanced pathological stages. Functional enrichment analyses further revealed that TUBA1B regulates key cell cycle processes, driving tumor proliferation, migration, and invasion. It also influences immune functions within both the innate and adaptive immune systems, affecting immune-related signaling pathways. These insights underscore TUBA1B’s multifaceted role in cancer progression and immune response.

**Conclusion:** This study highlights TUBA1B’s potential as a human oncogene with substantial roles in tumorigenesis and immune regulation. Elevated TUBA1B levels are associated with an immunosuppressive tumor microenvironment, impacting cancer progression and treatment outcomes. Targeting TUBA1B may offer promising therapeutic avenues for enhancing cancer treatment, offering new perspectives for innovative anti-tumor strategies with high clinical impact.

## Introduction

Malignant neoplasms pose a significant global health challenge, heavily impacting patients, families, and society as a whole (Sonkin et al., 2024). Despite substantial advancements in cancer therapies, the prognosis for many cancer types remains unsatisfactory (Zhang et al., 2020; Kandoth et al., 2013). Cancer treatment has evolved significantly over time. Traditional approaches such as surgery, chemotherapy, and radiotherapy have been the cornerstones, while emerging therapies like immunotherapy and targeted therapy are now revolutionizing the field, providing new hope and possibilities for patients. Within this complex landscape, the tumor microenvironment (TME) plays a critical role in driving tumor progression (Garassino et al., 2020). Tumor cells can extensively remodel the TME, influencing immune and stromal cells to create an environment conducive to uncontrolled tumor growth and spread (Nishino et al., 2017). Enhancing patient prognosis and quality of life requires a deeper understanding of the mechanisms through which the TME contributes to tumor progression.

Tubulin alpha 1b (TUBA1B), a protein-coding member of the human consensus coding sequence (Lin et al., 2020), is vital to the cytoskeleton and exists in five forms: α-, β-, γ-, δ-, and ε-tubulin. Tubulin is essential for maintaining cell shape, adhesion, motility, and regulating replication, division, mitosis, and intracellular transport (Kim et al., 2010; Reader et al., 2019). Microtubules, composed of α- and β-tubulin heterodimers, are dynamic structures integral to many cellular functions (Cook et al., 2020; Tuszynski et al., 2020). Although research on TUBA1B’s role in human cancers is limited, some studies have linked it to poor prognosis and chemotherapy resistance in hepatocellular carcinoma (Lu et al., 2013) and disease progression in Wilms’ tumor (Xu et al., 2020). However, a comprehensive pan-cancer analysis of TUBA1B is still lacking.

Aberrant activation of immune pathways in cancer involves abnormal immune signaling, leading to dysregulated immune cell activation and function (Zou et al., 2020). Tumor cells leverage this aberrant activation by releasing immunosuppressive factors to evade immune attacks, thus promoting growth and metastasis (Gajewski et al., 2013). This immune-suppressive tumor microenvironment is characterized by high numbers of immune-suppressive cells and molecules surrounding the tumor (Zou et al., 2020; Gajewski et al., 2013). Here, tumor cells activate signaling pathways in immune pathways, inducing immune cells to express immunosuppressive molecules, thereby inhibiting immune cell activation and limiting their attack on tumor cells (Jia et al., 2021). The increase in immune-suppressive cells exacerbates this immune-suppressive state. In response to such challenges, immunotherapy has become a widely studied and applied cancer treatment (Ivashkiv, 2018). The aim of immunotherapy is to reactivate the patient’s immune system, enabling immune cells to recognize and combat tumor cells. One of the most successful approaches is immune checkpoint inhibitors, which block inhibitory molecules on immune cells to restore their function and enhance their ability to target tumor cells (Nishino et al., 2017). Other methods, such as CAR-T cell therapy, tumor vaccines, and immune cell transfer therapy, aim to harness immune responses to control tumor growth (Zhang and Zhang, 2020). Combining immunotherapy with traditional treatments like chemotherapy, radiation, and targeted therapy offers a multi-pronged approach for enhanced treatment outcomes.

To gain a comprehensive understanding of TUBA1B’s involvement in various cancers, we conducted an extensive study, examining its expression patterns, genetic alterations, and prognostic relevance. Using multi-omics data across 33 different cancer types and advanced analytical techniques such as Gene Set Enrichment Analysis (GSEA) and Gene Set Variation Analysis (GSVA), we clarified TUBA1B’s role in tumor progression and its association with immune pathways (Ye et al., 2021; Liu and Tang, 2023b). Our findings reveal a strong association between elevated TUBA1B expression and an immunosuppressive tumor microenvironment, suggesting TUBA1B may be a predictive marker for immunosuppression within tumors. These insights provide a valuable understanding of TUBA1B’s potential role in cancer development, progression, and immune modulation.

## Materials and methods

### Data source

Clinical data and RNA sequences from 11,069 samples across 33 cancer types were gathered from the TCGA database using UCSC Xena (https://xena.ucsc.edu/). To compare these cancer samples with normal tissues, gene profile data from GTEx (https://commonfund.nih.gov/GTEx) provided a vital reference.

### Cell culture

A549, HBE, PC9, LLC, H1975, and H1299 cell lines were cultured in RPMI-1640 with 10% FBS and 1% penicillin-streptomycin at 37°C and 5% CO₂. Subsequently, the medium was replaced every 2–3 days to maintain optimal growth conditions and prevent nutrient depletion and metabolite accumulation. RNA was extracted using TRIzol Reagent, converted to cDNA via PrimeScript™ RT Reagent Kit, and analyzed by quantitative Real-time PCR (qPCR) with SYBR Premix Ex Taq II Reagent Kit. TUBA1B primers used were: forward 5′-CTC​AGT​TGA​TTA​TGG​CAA​GAA​GTC-3′ and reverse 5′-AGG​CGG​TTA​AGG​TTA​GTG​TAG​GT-3′. GAPDH was used as a reference. Relative expression was calculated by the 2^−ΔΔCT^ method, and analyses were performed in GraphPad Prism.

### EdU incorporation assay

Cell proliferation was measured with an EdU incorporation assay, where cells were incubated with EdU, fixed, and detected using the Click-iT EdU Imaging Kit. DAPI counterstained nuclei, and images were captured by fluorescence microscopy.

### Migration and invasion assays

Transwell assays were used to evaluate migration and invasion. For migration, transfected A549 cells were seeded in serum-free medium in the upper chamber, with medium containing 10% FBS in the lower chamber. After 24 h, migrated cells were fixed, stained, and counted. Invasion assays followed similar steps, but with Matrigel-coated inserts.

### CCK-8 assay

Cell viability was determined using the Cell Counting Kit-8 (CCK-8) assay. Cells were seeded into 96-well plates and incubated overnight at 37°C in a 5% CO₂ incubator to ensure cell attachment. The medium in each well was carefully aspirated and replaced with 100 μL of fresh medium containing 10% CCK-8 solution. The plates were then incubated for an additional 2 h at 37°C. Absorbance was measured at 450 nm using a microplate reader.

### Immunohistochemical staining

The Human Protein Atlas (http://www.proteinatlas.org/) provided immunohistochemical data on TUBA1B, allowing analysis of expression and localization patterns in tumors and normal tissues.

### Prognostic analysis

Using the TCGA database, we analyzed TUBA1B expression and cancer prognosis through survival indices: overall survival (OS), disease-specific survival (DSS), progression-free interval (PFI), and disease-free interval (DFI). Analyses were conducted with R’s “survival” and “forestplot” packages, using univariate Cox analysis to assess TUBA1B’s impact on these outcomes.

### GSEA and GSVA

Gene Set Enrichment Analysis (GSEA) assessed TUBA1B and associated genes in cancers using Pearson correlation and the R “clusterProfiler” package. Gene Set Variation Analysis (GSVA) was performed with “ssGSEA” and data from MSigDB.

### Tumor microenvironment analysis

We used the “ESTIMATE” R package to assess stromal, immune, and tumor purity scores in the TCGA cohort. Correlations between TUBA1B expression and TME-related pathways were explored, focusing on immune infiltration, immunomodulatory genes, MHC genes, and chemokines/receptors. Data from the Immune Cell Abundance Identifier (ImmuCellAI) and TIMER2 (http://timer.cistrome.org/) were visualized with the “ggplot2” package.

### Statistical analysis

Differential TUBA1B expression between cancer and normal tissues was assessed with t-tests. Specifically, for the *t*-test analysis, we first verified the assumptions of normality and homogeneity of variances for the data. If the data met the assumptions of parametric tests, an independent samples *t*-test was employed to compare the means of TUBA1B expression levels between the cancerous and normal tissue groups. In cases where the assumptions were violated, non-parametric alternatives were considered. Kaplan-Meier survival analysis and log-rank tests evaluated patient outcomes. Correlations were tested using Spearman’s or Pearson’s test, as appropriate. All analyses were performed in R (version 4.1.0), with p-values <0.05 considered significant, ensuring reliability in deriving conclusions.

## Results

### Expression of TUBA1B in human cancers

**FIGURE 1:**
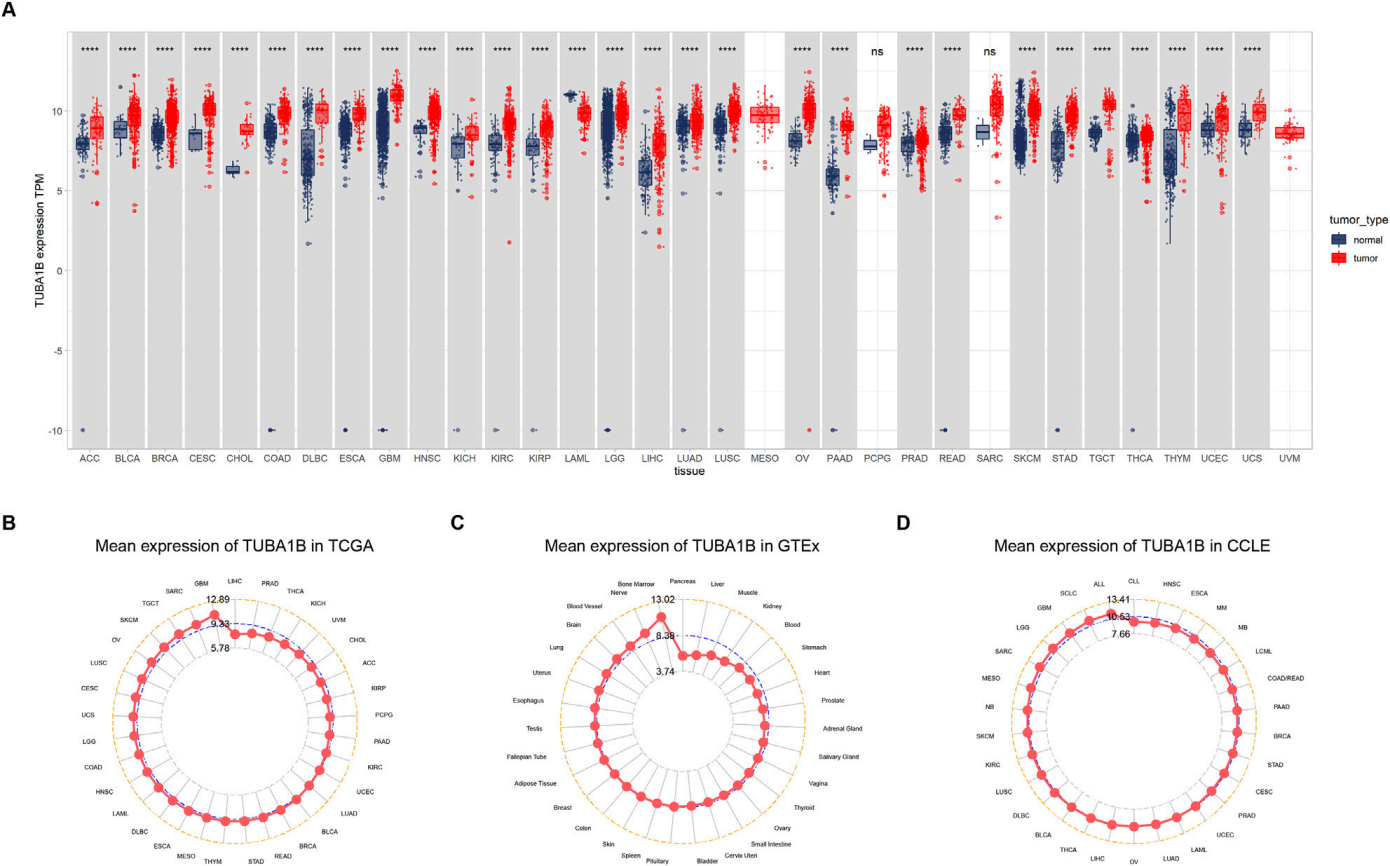
Expression of TUBA1B in pan-cancer. **(A)** Human cancers expression of TUBA1B. **(B)** TUBA1B expression in tumor tissues from TCGA cohort. **(C)** TUBA1B expression in normal tissues from GTEx cohort. **(D)** TUBA1B expression in cancer cell lines from the CCLE cohort. *P < 0.05, **P < 0.01, ***P < 0.001, ****P < 0.0001.

**FIGURE 2:**
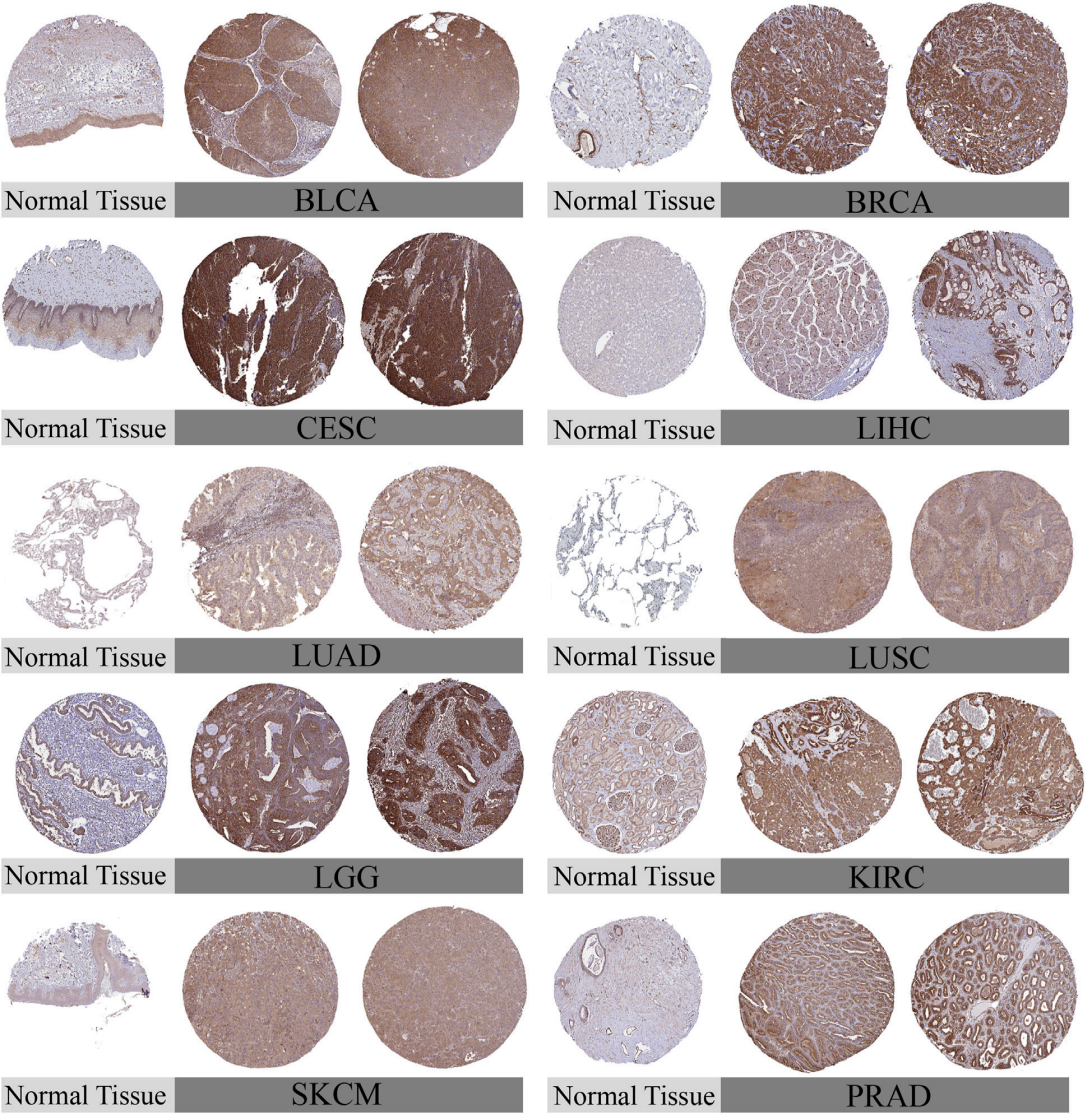
Representative immunohistochemical staining (IHC). The protein expression of TUBA1B in Bladder Urothelial Carcinoma, BLCA; Breast invasive carcinoma, BRCA; Cervical squamous cell carcinoma and endocervical adenocarcinoma, CESC; liver hepatocellular carcinoma, LIHC; Lung adenocarcinom, LUAD; Lung squamous cell carcinoma, LUSC; Lower Grade Glioma, LGG; Kidney Chromophobe, KIRC; Skin Cutaneous Melanoma, SKCM; Prostate adenocarcinoma, PRAD.

To comprehensively examine TUBA1B expression patterns in various human cancers, an extensive analysis was conducted using data from The Cancer Genome Atlas (TCGA) and Genotype-Tissue Expression (GTEx) databases. This investigation aimed to elucidate the TUBA1B mRNA expression levels across different cancers and compare them between normal and tumor tissues across multiple cancer types. The analysis revealed significant upregulation of TUBA1B in 28 out of the 33 cancers studied. Notably, cancers with increased TUBA1B expression included adrenocortical carcinoma (ACC), bladder urothelial carcinoma (BLCA), breast invasive carcinoma (BRCA), cervical squamous cell carcinoma and endocervical adenocarcinoma (CESC), cholangiocarcinoma (CHOL), colonic adenocarcinoma (COAD), diffuse large B-cell lymphoma (DLBC), esophageal cancer (ESCA), glioblastoma multiforme (GBM), head and neck squamous cell carcinoma (HNSC), renal chromophobe cell carcinoma (KICH), renal clear cell carcinoma (KIRC), kidney papillary cell carcinoma (KIRP), low-grade glioma (LGG), hepatocellular carcinoma (LIHC), lung adenocarcinoma (LUAD), lung squamous cell carcinoma (LUSC), ovarian plasmacytoid cystic adenocarcinoma (OV), pancreatic adenocarcinoma (PAAD), prostate adenocarcinoma (PRAD), rectal adenocarcinoma (READ), cutaneous melanoma (SKCM), gastric adenocarcinoma (STAD), testicular germ cell tumor (TGCT), thyroid adenocarcinoma (THCA), thymoma (THYM), endometrial cancer (UCEC), and uterine carcinosarcoma (UCS) (Figure 1A; Supplementary Table S1). Interestingly, acute myelogenous leukemia (LAML) exhibited lower TUBA1B expression, distinguishing it from other cancer types. Further comparative analysis of TUBA1B expression levels across tumor tissues showed that glioblastoma multiforme (GBM) had the highest expression, while hepatocellular carcinoma (LIHC) exhibited the lowest (Figure 1B). In normal tissues, TUBA1B expression was highest in bone marrow and lowest in the pancreas (Figure 1C). These expression patterns were validated through immunohistochemical data from the Human Protein Atlas (HPA), which showed strong TUBA1B staining in tumor tissues, whereas normal tissues exhibited weaker staining (Figure 2). These findings suggest TUBA1B’s potential as a biomarker for diagnostic and therapeutic applications. Further investigation into TUBA1B’s mechanisms and functions in these cancer types could pave the way for targeted interventions and personalized treatments, enhancing patient outcomes.

### Prognostic role of TUBA1B

**FIGURE 3:**
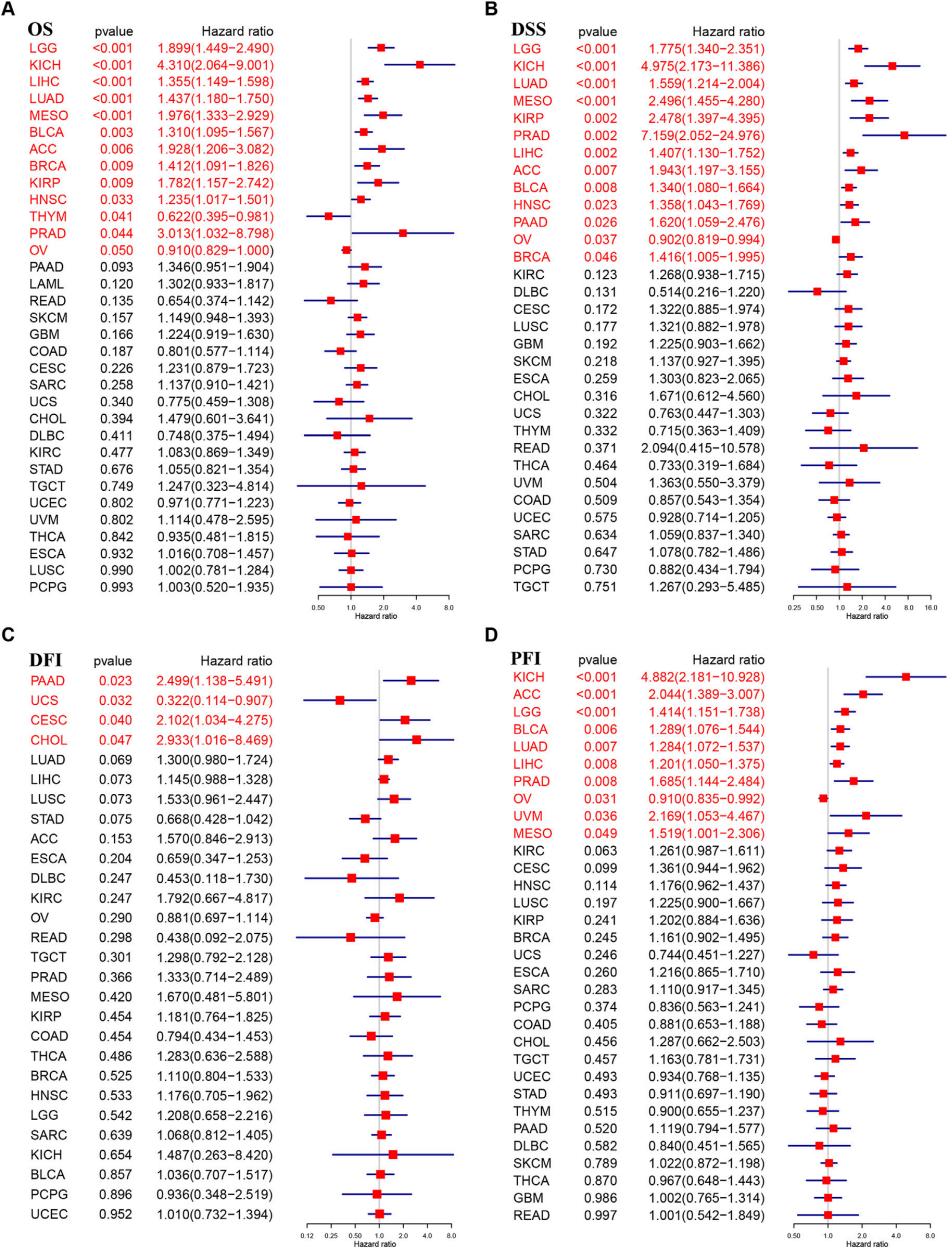
Prognostic value of TUBA1B. Forest plots showing results of Univariate Cox Regression analysis for **(A)** OS, **(B)** DSS, **(C)** DFI, and **(D)** PFI. Red color represents significant results (p < 0.05).

**FIGURE 4:**
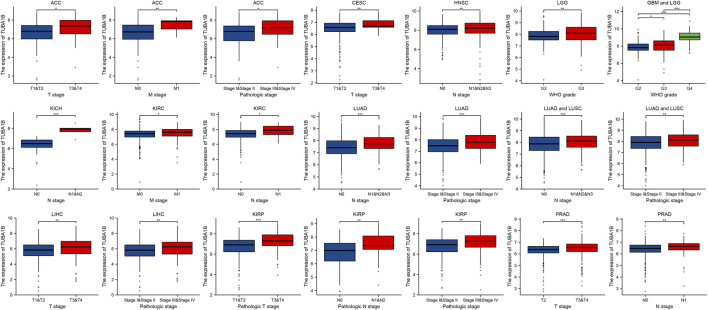
TUBA1B expression in different kinds of pathological stage. Differential analysis of TUBA1B expression in different kinds of pathological stages.

To understand TUBA1B’s role in cancer prognosis, an in-depth analysis was performed using univariate Cox regression to examine risk ratios for overall survival (OS), disease-specific survival (DSS), disease-free interval (DFI), and progression-free interval (PFI) across various cancers. This analysis revealed that high TUBA1B expression is a significant risk factor for OS in several cancers, including LGG, KICH, LIHC, LUAD, MESO, BLCA, ACC, BRCA, KIRP, HNSC, THYM, PRAD, and OV (Figure 3A, P < 0.05), suggesting that elevated TUBA1B expression correlates with poorer survival outcomes in these cancers. Similarly, TUBA1B was a risk factor for DSS in LGG, KICH, LUAD, MESO, KIRP, PRAD, LIHC, ACC, BLCA, HNSC, PAAD, OV, and BRCA (Figure 3B), underscoring its prognostic relevance. High TUBA1B expression was also associated with shorter disease-free intervals in patients with PAAD, UCS, CESC, and CHOL (Figure 3C), indicating a potential role in disease recurrence and progression. Cox regression analysis for PFI revealed that TUBA1B was an unfavorable factor in patients with KICH, ACC, LGG, BLCA, LUAD, LIHC, PRAD, OV, UVM, and MESO (Figure 3D), further supporting its use as a prognostic marker. Further analysis revealed that TUBA1B upregulation consistently correlated with advanced stages in various cancers (Figure 4), suggesting its role in tumor progression and aggressiveness and its potential as a marker for disease stage. These findings highlight TUBA1B as a critical prognostic indicator in cancer.

### Genetic alterations

**FIGURE 5:**
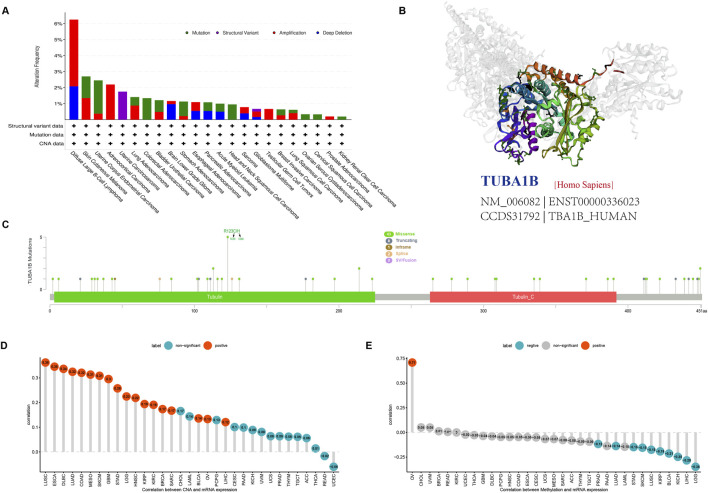
Gene alteration of TUBA1B. **(A)** The mutation and CNA status of TUBA1B in TCGA-human cancers. **(B)** The three-dimensional structure of TUBA1B protein. **(C)** Mutation site of the TUBA1B. **(D)** The correlation between TUBA1B expression and CNA. Red color represents significant results. **(E)** The correlation between TUBA1B expression and DNA methylation. Blue color represents significant results.

Tumorigenesis and cancer progression are closely associated with genetic changes, including gene mutations, copy number variants (CNV), and DNA methylation. To fully understand the genetic mutations affecting TUBA1B, we conducted an analysis of tumor samples from 10,953 patients using the cBioPortal platform. The three-dimensional structure of TUBA1B is shown in Figure 5A. Our findings reveal a high prevalence of TUBA1B amplifications, with diffuse large B-cell lymphoma showing the highest frequency (Figure 5B). Specific mutation sites and types in the TUBA1B gene are detailed in Figure 5C, highlighting its diverse mutation profile. In addition, a significant positive correlation between TUBA1B expression and CNV was identified across various cancers (Figure 5D), suggesting that CNV may drive TUBA1B dysregulation, influencing tumor progression. To further investigate, we analyzed DNA methylation’s role in TUBA1B expression, given its known impact on immune cell regulation and tumor immunosurveillance. This analysis across multiple cancers revealed significant correlations between TUBA1B expression and promoter methylation in nine tumor types, with ovarian cancer (OV) showing the highest positive and lower-grade gliomas (LGG) the highest negative correlation (Figure 5E).

### Biological function

**FIGURE 6:**
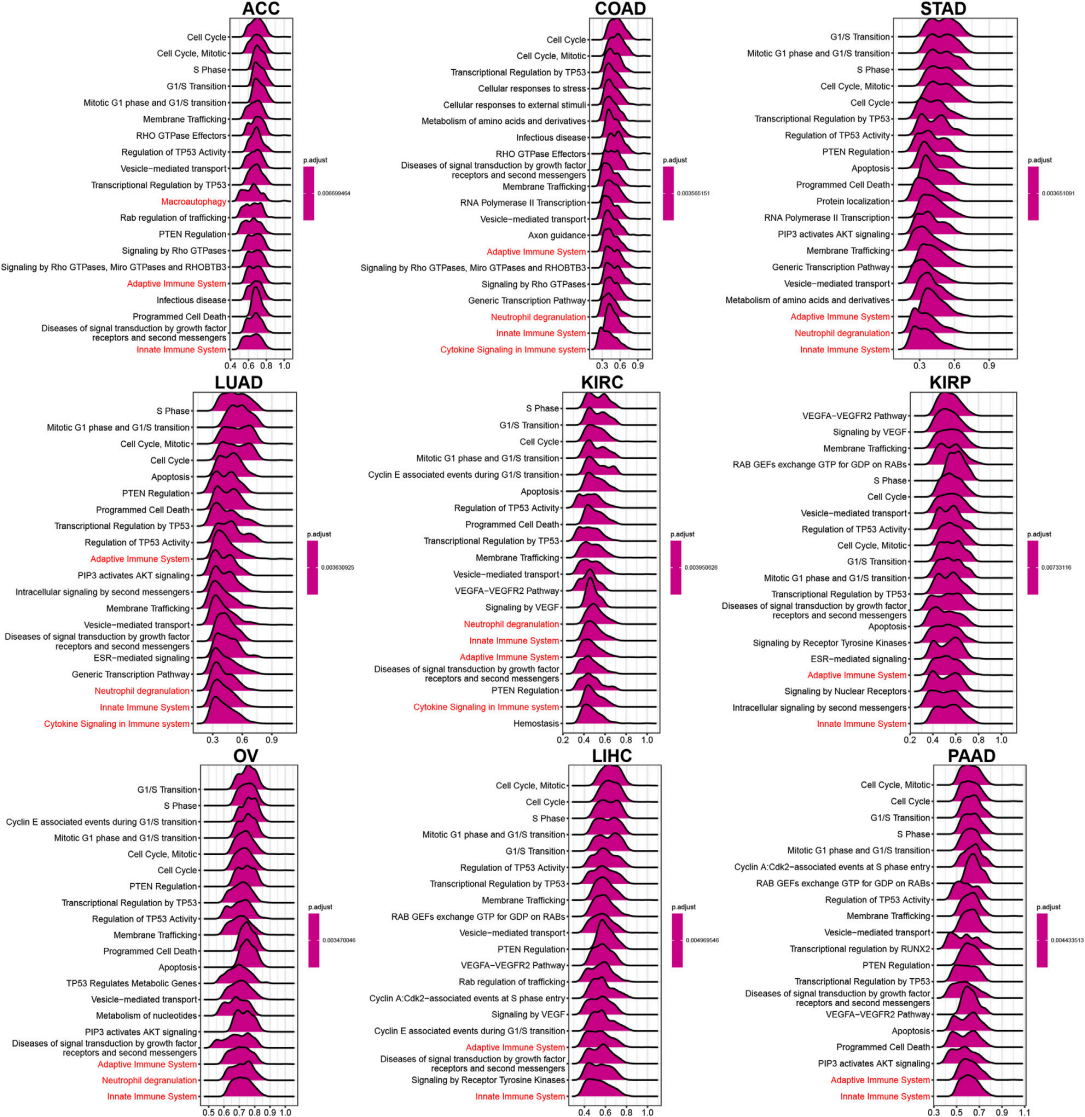
GSEA of TUBA1B. (A–I) The top 9 significant pathways of TUBA1B GSEA results across the indicated tumor types. Red color represents immune-related pathways.

To explore TUBA1B’s biological roles across cancers, we conducted a Gene Set Enrichment Analysis (GSEA) using the “clusterprofiler” package. This analysis revealed that TUBA1B is involved in cell cycle pathways, including “Cell Cycle,” “Signaling by Rho GTPases, Miro GTPases, and RHOBTB3,” and “Vesicle-mediated transport,” all found across nine cancer types (Figure 6). These pathways underscore TUBA1B’s regulatory role in fundamental cellular processes within cancer. Additionally, TUBA1B is engaged in immunomodulation pathways linked to adaptive and innate immunity and cytokine signaling, pointing to its dual involvement in tumor growth and immune system responses.

**FIGURE 7:**
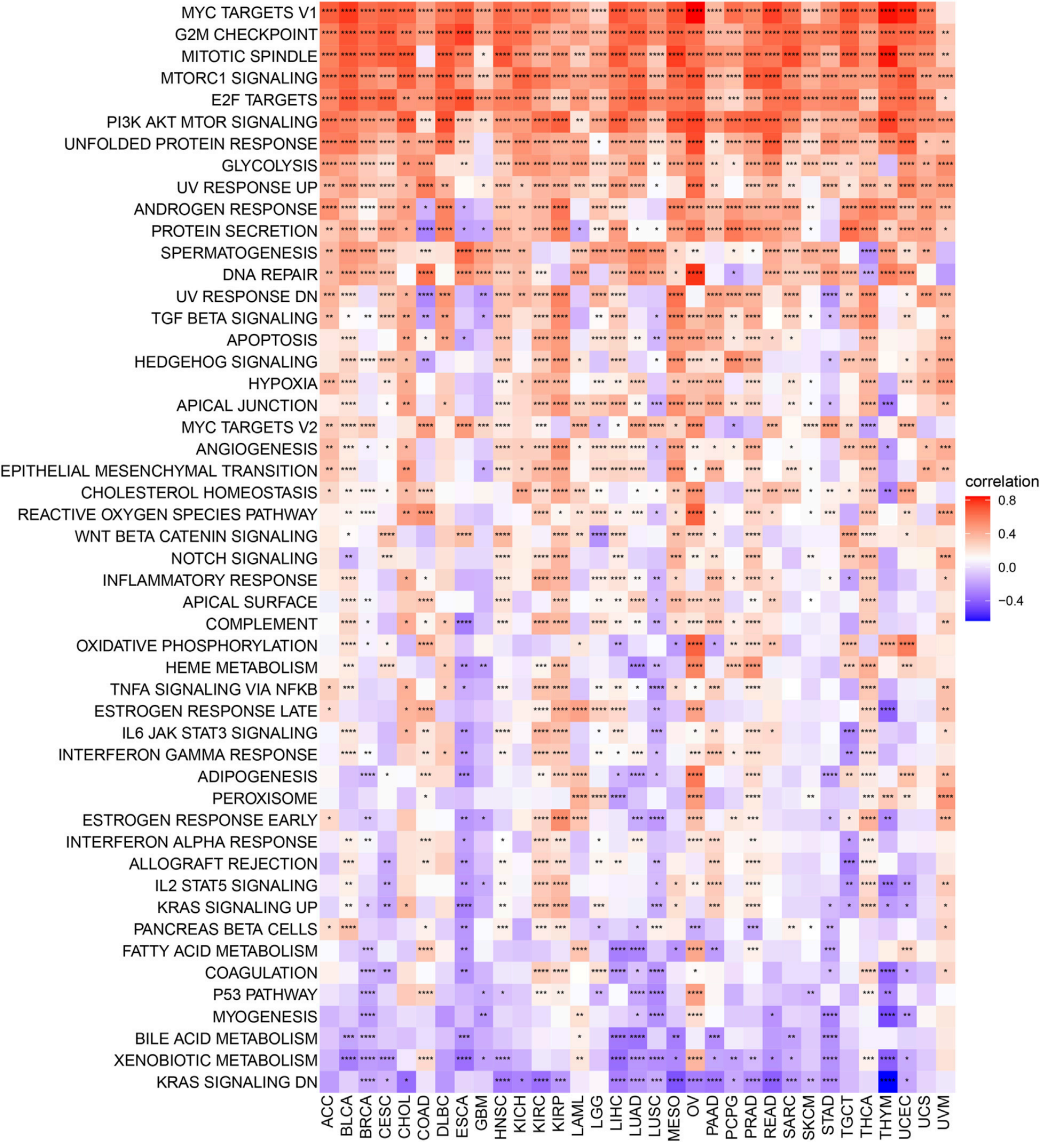
GSVA of TUBA1B. GSVA results of 50 hallmark pathways from the MSigDB.

Further, a comprehensive Gene Set Variation Analysis (GSVA) was conducted across 50 hallmark pathways (Figure 7), revealing a strong association between TUBA1B expression and pathways that drive cancer progression, such as hypoxia, KRAS, P53, and MYC signaling. This suggests TUBA1B’s influence on core molecular processes in tumor development. TUBA1B expression also positively correlated with immune-related pathways like interferon-alpha/gamma response, IL-2/STAT5 signaling, and IL6/JAK/STAT3 signaling, indicating TUBA1B’s significant role in the tumor immune microenvironment (TIME).

**FIGURE 8:**
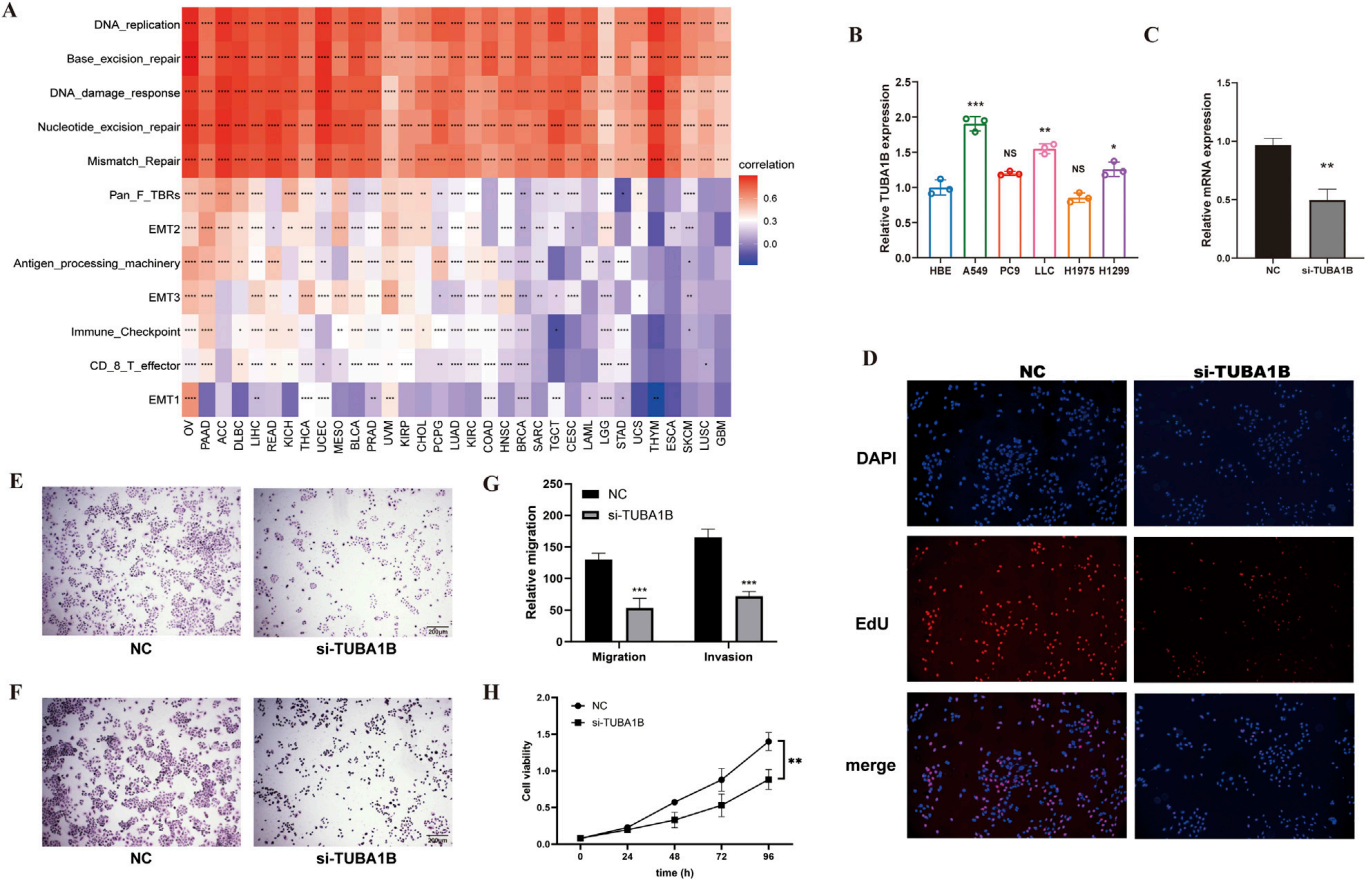
The Relationship Between TUBA1B, Cellular Processes, and Tumor Microenvironment Regulation **(A)** The correlation between TUBA1B and various cellular processes and the tumor microenvironment. Red indicates a positive correlation, blue indicates a negative correlation; the deeper the color, the stronger the correlation. **(B)** Expression levels of TUBA1B in various lung cancer cell lines. **(C)** Relative mRNA expression levels of TUBA1B in A549 cells after gene knockdown. **(D)** Fluorescence microscopy images showing the proliferation of A549 cells after TUBA1B knockdown. **(E)** Migration assay images showing the migration capability of A549 cells after TUBA1B knockdown. **(F)** Invasion assay images showing the invasion capability of A549 cells after TUBA1B knockdown. **(G)** Statistical analysis of the migration and invasion assays. **(H)** CCK-8 assay showing cell viability and proliferation after TUBA1B knockdown.*P < 0.05,**P < 0.01,***P < 0.001,****P < 0.0001.

To validate these findings, we examined TME-related pathways using TCGA immune data and a large-scale clinical study. Results confirmed TUBA1B’s close association with pathways involved in tumor proliferation and immune response. Figure 8A presents a heatmap showing correlations between TUBA1B and cellular processes in the TME. Red indicates positive and blue negative correlations, with intensity reflecting correlation strength. Data indicate that TUBA1B is positively associated with DNA replication, repair, and antigen processing and negatively correlated with immune checkpoint and CD8^+^ T cell functions, suggesting its dual role in promoting cell growth and suppressing immune responses. Figure 8B shows TUBA1B expression across various lung cancer cell lines, with notably higher expression in A549 and LLC cells compared to HBE (normal control). This upregulation in cancer cells may support their proliferation and survival. Knockdown of TUBA1B in A549 cells significantly reduced mRNA levels (Figure 8C), confirming the gene knockdown’s efficacy.

In a cell proliferation assay, fluorescence microscopy revealed numerous EdU-positive cells (red) in the control group, indicating active proliferation, while the si-TUBA1B group had fewer EdU-positive cells (Figure 8D), showing TUBA1B knockdown’s inhibitory effect on proliferation. Migration assay results showed more cells migrating through the Transwell membrane in the control group compared to the si-TUBA1B group (Figure 8E), suggesting that TUBA1B knockdown hinders cell migration. Similarly, invasion assays indicated that more cells invaded through the Matrigel in the control group compared to the si-TUBA1B group (Figure 8F), demonstrating that TUBA1B knockdown reduces cell invasion. Figure 8G is the statistical analysis of the migration and invasion results. In the CCK-8 assay, the results indicated a significant difference between the control group and the si-TUBA1B group. The control group exhibited a relatively high absorbance, while the si-TUBA1B group showed a notably lower absorbance, demonstrating that the knockdown of TUBA1B led to a marked reduction in cell viability and proliferation (Figure 8H), further confirming the role of TUBA1B in cell growth as detected by the CCK-8 assay. These findings underscore TUBA1B’s critical role in promoting cell proliferation, migration, and invasion, aligning its expression with oncogenic and immune-suppressive pathways. These results further support TUBA1B as an oncogene in cancer.

### Tumor microenvironment analysis

**FIGURE 9:**
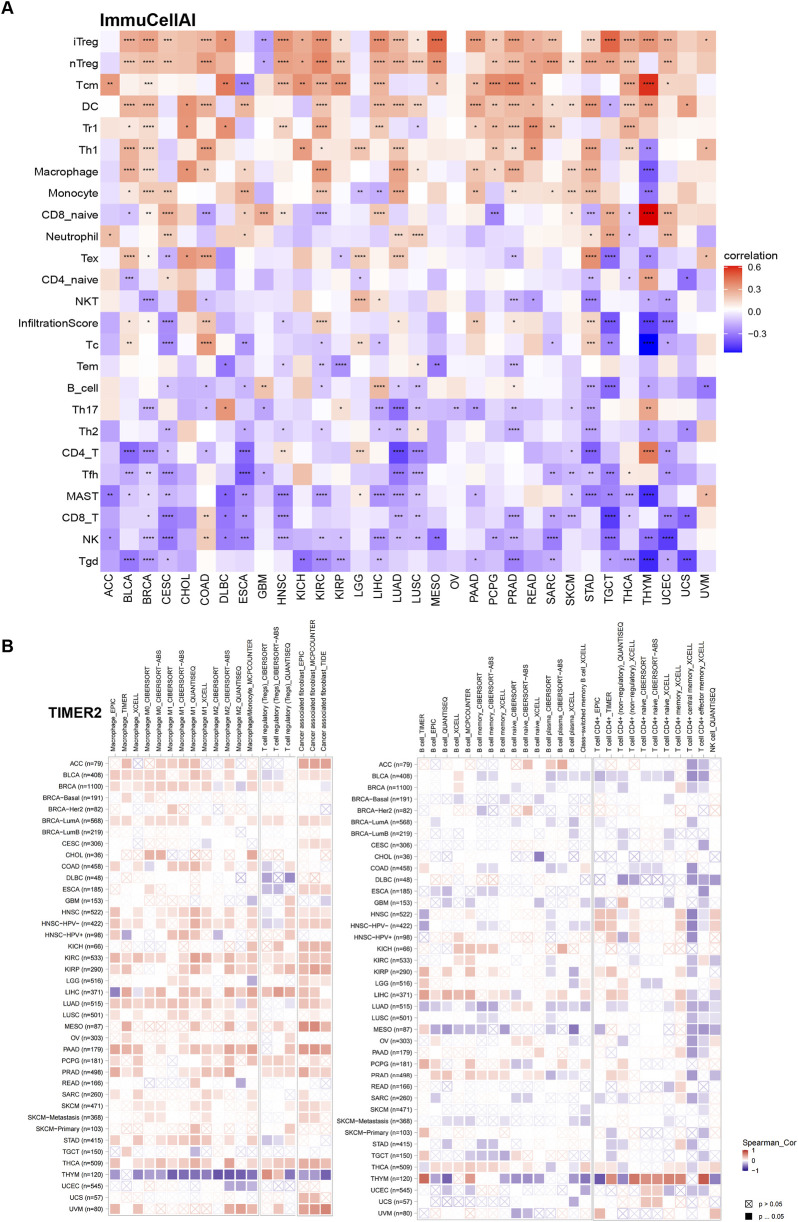
The relationship between TUBA1B and the immune cell infiltration. **(A)** Correlation between TUBA1B expression and different immune cells from ImmuCellAI database. **(B)** Correlation between TUBA1B expression and different immune cells from TIMER2 database. Red represents positive correlation, blue represents negative correlation, and the darker the color, the stronger the correlation. *P < 0.05, **P < 0.01, ***P < 0.001, ****P < 0.0001.

To deepen understanding of TUBA1B’s role in the immunosuppressive tumor microenvironment, we performed correlation analyses to examine TUBA1B expression, immune cell infiltration, and immune-related gene interactions. Immune data from ImmuCellAI and TIMER2 were analyzed independently (Figures 9A,B), consistently showing a positive correlation between TUBA1B expression and immunosuppressive cells like regulatory T cells (Tregs), tumor-associated macrophages (TAMs), and cancer-associated fibroblasts (CAFs). This strong positive correlation suggests that high TUBA1B expression contributes to an immunosuppressive TME. Conversely, TUBA1B expression negatively correlated with immune killer cells, including NK/NKT cells, CD4^+^ T cells, and CD8^+^ T cells, which are essential for anti-tumor immunity, indicating that elevated TUBA1B levels may inhibit effective immune responses.

**FIGURE 10:**
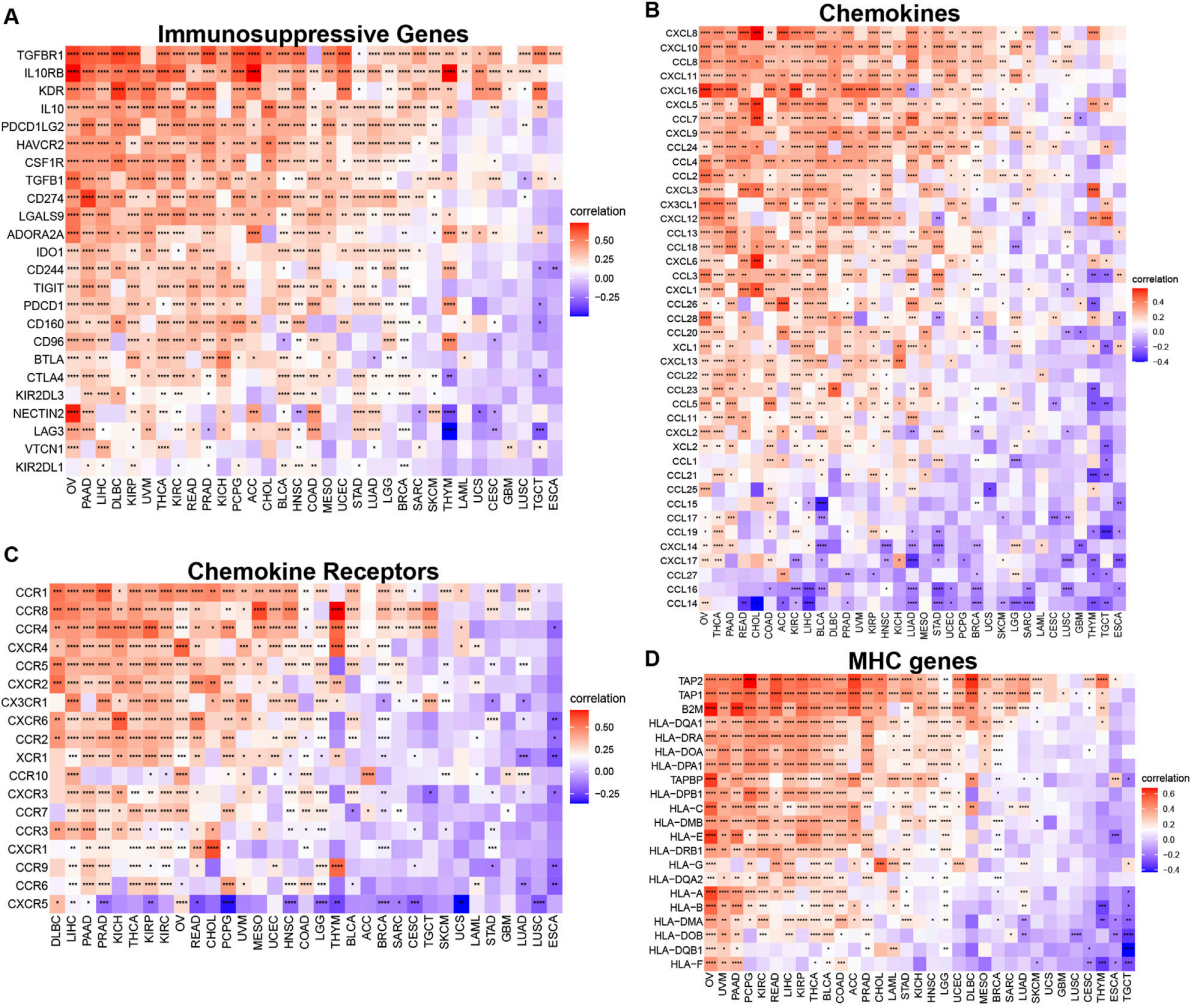
Relationship between TUBA1B expression and that of immune-related genes. **(A)** Immunosuppressive genes. **(B)** Chemokines. **(C)** Chemokine receptors. **(D)** MHC genes. Red represents positive correlation, blue represents negative correlation, and the darker the color, the stronger the correlation. *p < 0.05, **p < 0.01, ***p < 0.001.

Our analysis of TUBA1B’s influence on immune-related genes further revealed a positive correlation with key immunosuppressive genes, such as TIGIT, PDCD1 (PD-1), LAG3, CTLA4, and CD274 (PD-L1) (Figure 10A). These genes play vital roles in immune checkpoint regulation and cancer cell immune evasion, suggesting that TUBA1B may impact immunosuppressive factor expression. Furthermore, TUBA1B expression correlated positively with TGF-β and IL-10, two factors associated with TAMs and CAFs, indicating that TUBA1B may influence these cells’ function within the TME. Additional analysis revealed close associations between TUBA1B and immunomodulatory genes like chemokines (Figure 10B), chemokine receptors (Figure 10C), and MHC genes (Figure 10D). Key genes such as CCR2, CCL2, CXCR4, and CCR5 are critical for TAM recruitment, while MHC genes are essential for immune recognition and regulation. These analyses highlight TUBA1B as a central player in modulating the immunosuppressive TME. The observed positive correlations between TUBA1B and immunosuppressive factors and immune-regulatory genes provide insight into TUBA1B’s role in immune response interactions within cancer.

In conclusion, our study reveals the complex relationships between TUBA1B expression, immune cell infiltration, and immune genes within the TME. The positive correlations between TUBA1B and immunosuppressive factors suggest its potential role in establishing an immunosuppressive TME, offering valuable insights for developing targeted therapies to modulate the TME and enhance immune-based cancer control strategies for improved patient outcomes.

### The relationship of TUBA1B with immunotherapy response

**FIGURE 11:**
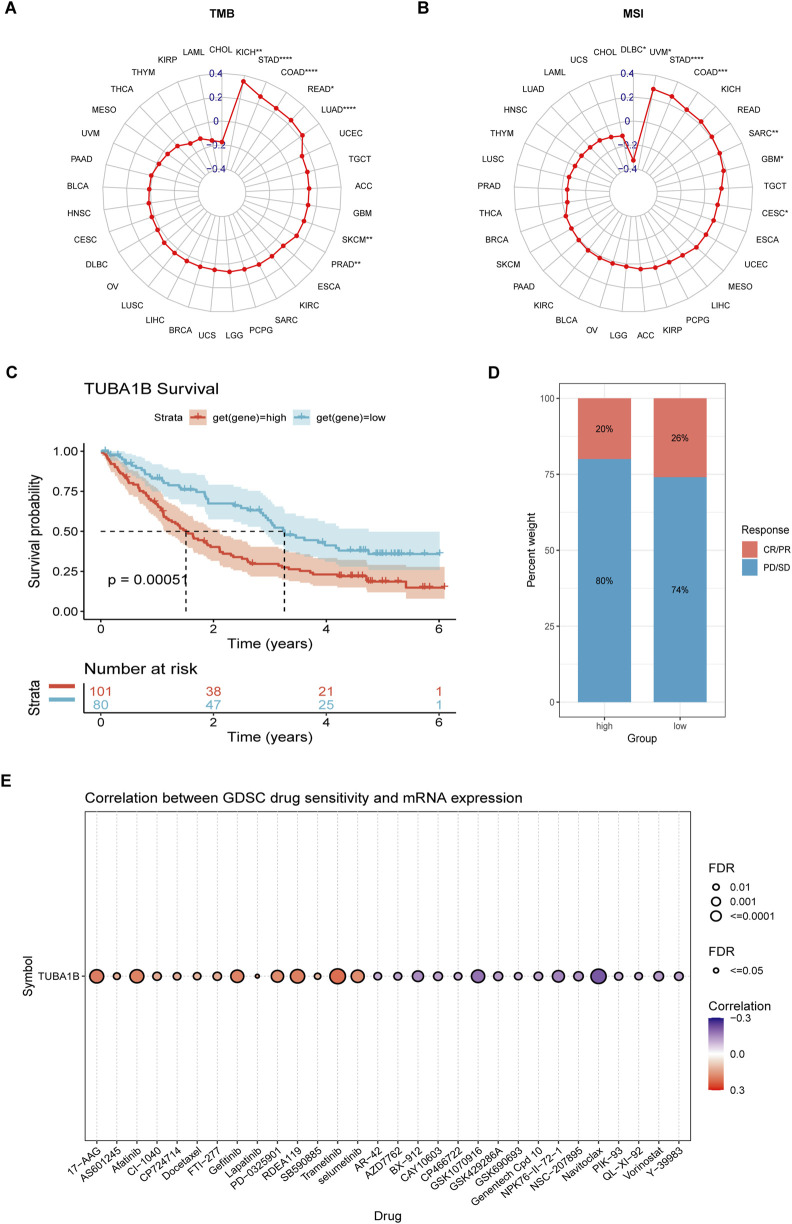
The association between TUBA1B expression and immunotherapy response. **(A, B)** Radar plot of the correlation between TUBA1B expression and TMB **(A)** and MSI **(B)**. **(C, D)** The Kaplan-Meier OS analysis **(C)** and percentage of responsive patients **(D)** in high- and low- TUBA1B expression groups of cohort (PMID:32472114). **(E)** The correlation between TUBA1B expression and the sensitivity of GDSC drugs (top 30) in human cancer. *P < 0.05, **P < 0.01, ***P < 0.001, ****P < 0.0001.

Recent advances in tumor immunotherapy, especially with immune checkpoint inhibitors (ICIs), have shown notable clinical success. Predictive biomarkers like tumor mutational burden (TMB) and microsatellite instability (MSI) scores are now established as indicators of response to ICI therapy and overall prognosis. In this study, we examined the relationship between TUBA1B expression and TMB/MSI across seven cancer types (Figures 11A, B). Our findings revealed significant correlations, suggesting that TUBA1B expression may strongly influence patients’ immunotherapy responses. To further validate these findings, we analyzed a comprehensive immunotherapy dataset, assessing the association between TUBA1B expression and clinical response across different cancers. Kaplan-Meier analysis demonstrated that low TUBA1B expression correlated with better outcomes, including extended overall survival and higher response rates in patients receiving ICIs (Figures 11C, D). These results indicate TUBA1B expression’s crucial role in determining patient response to immunotherapy.

Furthermore, we explored the Cancer Drug Sensitivity Genomics (GDSC) database, integrating IC50 values for 265 small molecule drugs with mRNA expression data from 860 cell lines, to identify potential antitumor drugs associated with TUBA1B expression (Figure 11E). The top 30 drugs showing the strongest correlations with TUBA1B expression reinforce its value as a predictive biomarker, while also expanding precision treatment options for lung adenocarcinoma patients. These insights underline the importance of considering TUBA1B expression in therapeutic planning. By leveraging the connection between TUBA1B expression and small molecule drugs, we unlock new avenues for targeted therapies tailored to individual patient profiles. Additionally, understanding the microenvironment effect on drug resistance in the context of TUBA1B dysregulation is crucial. The complex interplay between the tumor microenvironment and TUBA1B could potentially modulate the efficacy of therapeutic agents, highlighting the need for a more comprehensive approach that integrates both genomic and microenvironmental factors to overcome drug resistance and optimize treatment strategies, thus offering promising improvements in personalized cancer care and outcomes.

## Discussion

Tumorigenesis and cancer progression are highly complex processes involving dynamic interactions among various genes. Despite TUBA1B’s relevance as a key microtubule isoform, its significance in cancer has been largely underexplored (Lu et al., 2020; Xu et al., 2024). Although a few studies hint at TUBA1B’s potential as a cancer diagnostic marker, its broader role in cancer biology has not been fully understood (Lu et al., 2020). Thus, this study aimed to thoroughly examine TUBA1B’s expression, prognostic value, biological functions, and immunological implications in human cancers.

In this pioneering study, we analyzed TUBA1B expression and its prognostic importance across 33 cancer types, marking a first of its kind. We observed elevated TUBA1B levels in 28 cancers, which strongly correlated with advanced pathological stages and poorer prognoses, suggesting TUBA1B as a promising prognostic biomarker. Further investigation into gene mutations, copy number alterations (CNA), and DNA methylation’s influence on TUBA1B expression highlights its potential clinical value. Intriguingly, TUBA1B appears to engage in numerous immunomodulatory pathways, including those associated with adaptive and innate immunity, extending its role beyond cell structure maintenance. We noted that TUBA1B overexpression is linked to key immune-related pathways in the tumor immune microenvironment (TIME), such as IL-6/JAK/STAT3 signaling, IL-2/STAT5 signaling, TGF-Beta signaling, and interferon responses (Xu et al., 2024). These findings suggest TUBA1B as a significant player in modulating the immune landscape within tumors.

The tumor microenvironment (TME) is critical in cancer development, with the TIME playing a particularly pivotal role (Hartupee et al., 2024). While targeting TIME for cancer therapy has shown promise, concerns exist about its potential to support tumor growth and reduce immunotherapy efficacy. Immune cells within the TME, especially tumor-associated macrophages (TAMs), are major TIME contributors (Chu et al., 2024). TAMs typically present two phenotypes: pro-inflammatory (CAMs) that support immune responses against tumors and anti-inflammatory (AAMs) that promote immunosuppression through cytokines like IL-10 and TGF-β. The TME tends to polarize TAMs toward the AAM phenotype, creating an immunosuppressive environment that limits antitumor immunity. Additionally, regulatory T cells (Tregs) accumulate within the TME, facilitating immune evasion by malignant cells and impairing CD8^+^ T cell responses. The interplay between TAMs, Tregs, and tumor cells shapes an immunosuppressive TME, enabling tumor growth and metastasis. Understanding this complex network is essential for developing effective therapies. Targeting immunosuppressive pathways and modulating TAM and Treg activity could improve immunotherapy outcomes by counteracting the TME’s suppressive effects.

To evaluate the role of TUBA1B in shaping the immunosuppressive TME, we analyzed its correlations with immune-related genes (Liu et al., 2024a; Liu et al., 2024b). Results revealed significant associations between TUBA1B expression and immunosuppressive checkpoints, including PD-1, PD-L1, CTLA-4, LAG3, and TIGIT. Moreover, TAM recruitment was found to be influenced by immune-related genes such as CCL2, CCR2, CXCR4, and CCR5, which suppress immune responses and facilitate cancer progression. Additionally, Treg activation further exacerbates immunosuppression by enhancing the expression of checkpoints (e.g., PD-1, CTLA-4, TIM-3, TIGIT) and upregulating molecules like CD39, CD73, and CCR4, contributing to T cell dysfunction and altered trafficking. Together, these findings emphasize TUBA1B’s pivotal role in establishing an immunosuppressive TME, offering critical insights for therapeutic strategies aimed at reversing immunosuppression and improving anti-tumor immune responses. Our study also highlights TUBA1B’s involvement in mechanisms underlying drug resistance mediated by the TME. By identifying its role in modulating the tumor microenvironment and its connection to resistance pathways, we provide a novel perspective for developing combination therapies. This work not only advances understanding of microenvironment-mediated resistance but also lays the foundation for designing innovative therapeutic agents targeting the TUBA1B-microenvironment axis, ultimately improving cancer treatment outcomes.

Although our study provides significant insights, it has limitations, primarily due to reliance on publicly available data. Further *in vitro* and *in vivo* studies and large-scale prospective studies are required to validate our findings. Future research should build upon and explore these results in greater detail.

In conclusion, our findings highlight TUBA1B’s potential as a prognostic biomarker and therapeutic target in human cancers. High TUBA1B expression may foster an immunosuppressive TME, and combining TUBA1B targeting with immune checkpoint inhibitors could offer substantial therapeutic benefits. This study is the first to explore TUBA1B’s potential in tumor immunity, offering a novel perspective for advancing anti-tumor strategies.

## Data Availability

The original contributions presented in the study are included in the article/Supplementary Material, further inquiries can be directed to the corresponding authors.

## Glossary

AML: Acute myeloid leukemia
ACC: Adrenocortical carcinoma
BLCA: Bladder urothelial carcinoma
BRCA: Breast invasive carcinoma
CAFs: Cancer-associated fibroblasts
CCK-8: Cell counting kit-8
CHOL: Cholangiocarcinoma
COAD: Colon adenocarcinoma
CNV: Copy number variation
DFI: Disease-free interval
DSS: Disease-specific survival
ESCA: Esophageal squamous cell carcinoma
GSEA: Gene-set enrichment analysis
GSVA: Gene-set variation analysis
GBM: Glioblastoma multiforme
HNSC: Head and neck squamous cell carcinoma
ICIs: Immune checkpoint inhibitors
KICH: Kidney chromophobe
KIRC: Kidney renal clear cell carcinoma
KIRP: Kidney renal papillary cell carcinoma
LIHC: Liver hepatocellular carcinoma
LGG: Lower grade glioma
LUAD: Lung adenocarcinoma
LUSC: Lung squamous cell carcinoma
DLBC: Lymphoid neoplasm diffuse large B-cell lymphoma
MSI: Microsatellite instability
OS: Overall survival
OV: Ovarian serous cystadenocarcinoma
PCPG: Pheochromocytoma and paraganglioma
PRAD: Prostate adenocarcinoma
PFI: Progression-free interval
READ: Rectum adenocarcinoma
SSR3: Signal sequence receptor subunit 3
SKCM: Skin cutaneous melanoma
STAD: Stomach adenocarcinoma
TGCT: Testicular germ cell tumor
HPA: The Human Protein Atlas
THYM: Thymoma
THCA: Thyroid carcinoma
TAMs: Tumor-associated macrophages
TANs: Tumor-associated neutrophils
TME: Tumor microenvironment
TIME: Tumor immune microenvironment
TMB: Tumor mutational burden
UCS: Uterine carcinosarcoma
